# A note from the Editor-in-Chief: a time of gratitude

**DOI:** 10.1016/j.igie.2026.02.001

**Published:** 2026-03-30

**Authors:** Linda S. Lee

**Affiliations:** Brigham and Women’s Hospital and Harvard Medical School, Boston, Massachusetts, USA

We are absolutely thrilled to share that *iGIE* has been accepted for indexation in PubMed. This is a tremendous milestone for our young journal, and what a wonderful way to ring in 2026!

*iGIE* began just over 3 years ago, thanks to the incredible vision and support of the American Society for Gastrointestinal Endoscopy (ASGE) and the leadership of Dr Chris Thompson as the inaugural Editor-in-Chief. As ASGE's newest online-only journal, *iGIE* is dedicated to innovation, investigation, and insights related to gastrointestinal endoscopy. It offers a forum for early preclinical and clinical studies, interesting cases, historical and industry insights, as well as showcases interdisciplinary care across our health care providers and work from around the globe, including guidelines, consensus statements, reviews, and clinical discussions with geographic relevance outside the United States. We are incredibly proud of how far *iGIE* has come in such a short time.

I wanted to personally thank our entire team, as well as all the authors who entrusted us with their outstanding work, especially as a new journal. I am deeply indebted to the amazing trio of Stephanie Kinnan, Sarah Trotto, and Becca Meier from our Editorial Office, who truly make the journal possible and without whom PubMed indexation would have remained only a dream. They tirelessly shepherd all of us, corralling us along the straight and narrow path even as some of us stray at times (including myself!), to ensure each issue gets published in a timely manner and every article receives the thoughtful attention it deserves.

I am also incredibly grateful to our team of extraordinary Senior Associate and Associate Editors. Their enthusiasm for *iGIE* and daily commitment to treating every submission with the same care they would want for their own work have been foundational to our success. I am continually inspired not only by their remarkable expertise, dedication, and passion as world-leading endoscopists and researchers, but also by their creativity and commitment to growing our journal.

Our editors rely on our incredible reviewers and our newly formed Editorial Review Board, whose generosity and dedication make *iGIE* possible. This remarkable community of colleagues guides each manuscript from submission to final decision with care, rigor, and thoughtfulness. We are deeply grateful to all our reviewers for the time, care, and insight you so generously share amid your demanding schedules to elevate each article and help shape the voice and quality of each issue. We truly could not do this work without you.

Of course, none of this would have been possible without all our amazing authors. Thank you to every author who took a leap of faith and submitted your precious works to us from day 1, and for your patience as we learned and grew as a journal. We are all enormously indebted to you for your dedication to providing outstanding care for your patients, advancing our field, and sharing your unique insights with all of us, which we know is not an easy journey. We look forward to publishing many more extraordinary articles from all of you around the world!

My sincere thanks to Rachael Engels, Senior Publisher at Elsevier, and to the entire Elsevier team for being such incredible partners throughout this journey. I am profoundly grateful for your unwavering support, belief in *iGIE*, and the remarkable space you provided us with to grow over the past 3 years until our PubMed indexation became a reality. Your expertise and thoughtful guidance in this world of publishing have been invaluable in helping us reach this milestone.

It has truly been a privilege to work alongside such an outstanding community, and I look forward to a fantastic year ahead.

Gratefully,

